# Improving the oncology care pathway through the experience of patients and their caregiver

**DOI:** 10.1186/s12904-025-01839-7

**Published:** 2025-07-12

**Authors:** Stéphanie Gentile, Bilel Zidi, Andrée Robaglia-Schlupp, Romain Spetidi, Hanna Bouida, Elodie Cretel

**Affiliations:** 1 Aix Marseille Univ, School of medicine - Aix-Marseille Université, SSA, RITMES, , 2 Service d’Evaluation Medicale, Hôpital de La Conception, Assistance Publique des Hôpitaux de Marseille, 147 Boulevard Baille, Marseille, 13005 France; 2 Structure régionale d’appui à la qualité et la sécurité des soins, Provence-Alpes-Côte d’Azur, Marseille, France; 3 Contributeur indépendant, aidant familial / pair-aidant, Marseille, France; 4 Dispositif Spécifique Régional du Cancer pour les régions de Provence Alpes Côte d’Azur et de Corse, Marseille, France; 5 Agence Régionale de Santé Provence Alpes Côte d’Azur, Marseille, France

**Keywords:** Cancer care pathway Patient experience, Family caregivers, Oncology care coordination, Supportive care Qualitative research

## Abstract

**Purpose:**

This study aims to explore the lived experiences of individuals undergoing cancer treatment and their family caregivers to identify gaps and potential improvements in the oncology care pathway. It seeks to address the challenges faced during diagnosis, treatment, remission, and the transition to palliative care, emphasizing the need for patient-centered care.

**Methods:**

A qualitative exploratory study was conducted through focus groups with cancer patients (PCs) and family caregivers (FCs). Participants were recruited using a snowball method via oncology networks and patient associations. Six focus groups were held, with participants sharing their experiences across the care continuum. Data were collected using an open-ended interview guide, transcribed verbatim, and analyzed thematically to identify recurring themes and insights.

**Results:**

A total of 34 participants (25 PCs and 9 FCs) highlighted key challenges, including a lack of care coordination, insufficient communication, and limited access to supportive oncology services. Many PCs felt overwhelmed by administrative burdens, delays in testing, and inadequate follow-up care. FCs often reported feeling excluded from critical consultations and unsupported in caregiving responsibilities. Positive experiences were associated with coordinated care and access to supportive services, though these were inconsistently provided.

**Conclusion:**

The study underscores significant gaps in oncology care, particularly in care coordination, emotional support, and communication. Incorporating the voices of PCs and FCs into healthcare policies can foster a more empathetic, coordinated, and patient-centered oncology care system.

## Introduction

Almost every family in the world is affected by cancer, either directly or indirectly. One in five individuals will receive a cancer diagnosis during their lifetime, and the consequences of this disease extend well beyond the individual, impacting caregivers and family members as well [1]. According to Dr. Tedros Adhanom Ghebreyesus, Director-General of the World Health Organization (WHO), "a cancer diagnosis has vast and profound repercussions on the health and well-being of all those involved. For too long, the fight against cancer has been focused solely on clinical care and not on the broader needs of people affected by cancer" [2]. This statement underlines the urgent need to move beyond a purely biomedical approach and consider the emotional, social, and practical challenges faced by individuals living with cancer and their families.

Global cancer control policies should integrate not only data and scientific research but also the lived experiences of those directly affected [3]. Patient experience is increasingly recognized as a cornerstone of quality care. It encompasses not just clinical interactions, but also pre-consultation exchanges, hospitalization processes, and discharge planning [4]. It requires viewing the individual not solely through the lens of illness but as a whole person, with their personality, expectations, and needs [5]. Unlike satisfaction surveys—which often adopt a top-down logic and solicit opinions on preselected topics without follow-up—patient experience is grounded in the person’s perspective, informed by their trajectory, values, and concerns [6].

Understanding and giving greater visibility to the experiences of individuals living with cancer and their caregivers is essential to creating more responsive and supportive healthcare systems. However, these experiences remain underrepresented in decision-making processes.

France, with its 68.29 million inhabitants and 13 administrative regions, provides universal health coverage and implements national cancer strategies through structured care pathway [7]. These are deployed regionally through dedicated oncology networks. In the south-east of France, the Provence-Alpes-Côte d’Azur (PACA) and Corsica regions are covered by a joint regional oncology network (*OncoPACA-Corse*), which supports coordination across care providers. Within hospitals, Cancer Coordination Centers (3Cs) ensure the implementation of these pathways at the institutional level.

Despite these institutional efforts, individuals living with cancer and their caregivers frequently report difficulties navigating the system and accessing appropriate support. In fact, a recent national survey found that 41% of French people feel their goals and expectations are not sufficiently considered by the healthcare system [7]. This gap highlights the need to investigate how the oncology care pathway is experienced in practice, particularly in the PACA and Corsica regions.

As part of the Ten-Year Strategy for Fighting Cancer 2021–2030 (*Stratégie décennale de lutte contre les cancers*, 2021–2030) [8], the Regional Health Agency (ARS) of PACA launched a qualitative study to gather insights from individuals who have or have had cancer and their caregivers, regardless of cancer type. The aim was to document their lived experiences along the care pathway to identify areas for improvement.

In this study, the term *oncology care pathway* refers to the formal sequence of care services organized by the French healthcare system to ensure access, coordination, and support—from diagnosis to end-of-life care. It also reflects the subjective reality of those who live through it. This dual perspective highlights the potential misalignments between planned structures and actual lived experiences.

## Methodology

### Research design and approach

A qualitative exploratory study using focus groups was conducted in 2022 with individuals living with cancer, whether undergoing treatment, in remission, or recovering. Focus groups were chosen to encourage open discussion and explore both collective and individual experiences in a dynamic setting [9]. All steps of the study were co-developed with patient associations, the regional oncology network OncoPACA-Corse, and local Cancer Coordination Centres (3Cs) based in hospitals.

### Participants and sampling

Participants were individuals aged 18 and over who were currently being treated for cancer or had completed treatment (referred to as individuals with cancer), and their family caregivers. A snowball sampling method [10] was used to recruit participants through regional oncology stakeholders, including patient associations and 3Cs in the PACA-Corse region (South-East France, approximately 5 million inhabitants, with both urban and rural areas). These stakeholders used posters and emails to circulate recruitment information. Interested individuals registered through a QR code or internet link.

To ensure a broad range of perspectives and to capture the full continuum of the cancer care experience, focus groups were deliberately composed of participants with diverse profiles (in terms of age, gender, cancer type, and stage of disease). This heterogeneity was intended to enrich the discussions and help identify cross-cutting themes in patient and caregiver experiences. We adopted a differentiated organizational approach: four focus groups were conducted separately, with either persons with cancer (PCs) or family caregivers (FCs). This separation aimed to ensure greater freedom of expression for caregivers, as highlighted by several partner associations. Indeed, the presence of the person being cared for can sometimes inhibit caregivers from openly expressing certain difficulties. Two additional mixed focus groups were conducted, bringing together both patients and caregivers, to explore relational dynamics and shared or contrasting perceptions.

#### Data collection

Participants received written and oral information about the study in advance, including the objective, procedures, confidentiality, and their right to withdraw at any time. Participation was based on informed non-opposition, as per French regulations and ethics approval (Aix-Marseille University Ethics Committee, Ref: 2023–02-09–011).

The focus groups were conducted in French by the same experienced qualitative researcher (SG), with a second researcher (BZ) present to take notes and support facilitation. A semi-structured discussion guide was developed in consultation with patient associations and 3Cs, based on a central open-ended question:

*“Could you describe your experience, or that of the person you care for, throughout the cancer care pathway?”*

This guide covered the main stages of the “oncology care pathway” structured by the National Cancer Institute (INCa), which is composed of several coordinated phases [7]. It begins with the diagnostic phase, which includes initial assessments, confirmatory tests, and the mandatory disclosure consultation (*consultation d’annonce*), during which the diagnosis is communicated to the patient along with personalized information and support. This is followed by multidisciplinary treatment planning, during which patient cases are reviewed by a Multidisciplinary Tumor Board (*Réunion de Concertation Pluridisciplinaire*, RCP) to determine an individualized treatment strategy. The treatment phase encompasses the administration of medical interventions such as surgery, chemotherapy, radiotherapy, or targeted therapies. Throughout the care trajectory, supportive care (*soins oncologiques de support*) is offered to address physical, psychological, and social needs. After active treatment, patients enter the post-treatment follow-up phase, which includes surveillance, management of late effects, and support for reintegration into daily life. When needed, the final stage of the pathway is the integration of palliative and end-of-life care, focusing on comfort, dignity, and quality of life. This guide was the same for PCs and FCs.

The guide which also served as an initial coding framework. It was designed to support free expression while ensuring consistency across sessions.

Sociodemographic information was collected beforehand (e.g., cancer type/status, age, gender, location, employment) to contextualize the findings. All sessions were audio-recorded with consent. A summary of each focus group, based on transcripts, was shared with participants for feedback to ensure their perspectives were accurately captured. Quotes used in this paper were translated into English by the research team.

#### Data analysis

Recordings were transcribed verbatim, and transcripts were anonymized. The audio files were deleted after transcription, and transcripts were reviewed for completeness and accuracy by SG and BZ. Data were analyzed thematically following Braun and Clarke’s method [11], combining inductive and deductive approaches. Deductive categories were based on the stages of the care trajectory; inductive themes emerged from repeated readings of the transcripts.

Both SG and BZ independently conducted coding, then compared and discussed their analyses to resolve discrepancies. An initial coding framework was co-constructed and refined through iterative team discussions. No qualitative software was used.

One of the co-authors (ARSch), who also participated in a focus group as a caregiver, contributed to the thematic analysis and manuscript writing as part of a participatory research approach. This involvement was disclosed to other participants, and ARSch did not access any data from focus groups in which she was not involved.

For the presentation of the results, we will use the term 'participants' when statements are reported by PCs and FCs.

## Results

25 PCs and 9 FCs participated in the six focus groups. The characteristics of the participants are presented in Table 1. No individuals who attended the focus groups declined to participate, and none of those who had initially agreed to take part cancelled their participation. Despite variations in the size and composition of the focus groups, data saturation was achieved. This diversity allowed for the emergence of recurrent and complementary themes, reflecting the plurality of experiences across the cancer care pathway.

**Table 1:** Characteristics of the participants

P / FC	Age Group	SEX	ORGAN	Focus group number
P1	40–44	Female	OVARY	Focus 1
P2	50–54	Female	LUNG	Focus 1
P3	70–76	Male	MYELOMA	Focus 1
P4	80–84	Male	PROSTATE	Focus 1
P5	45–49	Female	MELANOMA	Focus 2
P6	45–49	Female	BREAST	Focus 2
P7	45–49	Female	BREAST	Focus 2
P8	45–49	Female	LUNG	Focus 2
P9	45–49	Female	BREAST	Focus 2
FC1	50–54	Female	MYELOMA	Focus 3
FC2	70–74	Female	BLADDER	Focus 3
FC3	45–49	Female	GLIOBLASTOMA	Focus 3
FC4	50–54	Female	PANCREAS	Focus 3
FC5	65–69	Man	KIDNEY	Focus 3
FC6	45–49	Female	STOMACH	Focus 3
FC7	40–44	Female	PROSTATE	Focus 3
FC8	45–49	Male	ACUTE LEUKEMIA	Focus 3
P11	45–49	Female	BREAST	Focus 4
P12	35–39	Female	BREAST	Focus 4
P13	70–75	Female	BREAST	Focus 4
P14	50–54	Female	RECTUM	Focus 4
P15	65–69	Male	PROSTATE	Focus 4
P16	50–54	Female	THROAT	Focus 4
P17	60–64	Female	BREAST	Focus 4
P18	50–54	Female	BREAST	Focus 4
P19	50–54	Female	BREAST	Focus 4
P20	40–44	Female	BREAST	Focus 4
P21	55–59	Female	ACUTE LEUKEMIA	Focus 4
FC9	60–64	Male	KIDNEY	Focus 5
P10	50–54	Female	BREAST	Focus 5
P22	55–59	Female	PANCREAS	Focus 6
P23	55–59	Female	BREAST	Focus 6
P24	45–49	Female	BREAST	Focus 6
P25	50–54	Female	BREAST	Focus 6

PC = people aged 18 and above who were being treated for cancer or who had been treated for cancer

FC = family caregivers

### Stage 1 diagnostic phase and announcement of cancer diagnostic workup and diagnostic disclosure consultation

This phase was described by participants as emotionally intense, marked by anxiety, fear, anger, denial, and sometimes despair.

#### Diagnostic phase: the psychological impact of test tesults

Several PCs reported that their cancer was detected incidentally during complementary tests, particularly imaging. As a result, some individuals suspected or even knew about their diagnosis before it was formally announced by a healthcare professional. This premature awareness, often lacking any accompanying explanation, generated heightened anxiety and uncertainty.

“*I saw the scan before the doctor spoke to me… I understood immediately. It was terrifying because no one had said anything yet*.” (Patient, FG2).

“*The doctor I saw told me that we had to wait for the biopsy results. Except that on the report, it was written ACR 5, and like all patients who have a report with things they don't understand, because even though we are healthcare workers, we don't understand everything. I looked it up in the elevator, to find out what an ACR 5 was. And ACR 5, it was cancer, confirmed*.” (Patient, FG2).

#### Experiencing the cancer diagnosis disclosure consultation

Most participants expressed dissatisfaction with the consultation during which the diagnosis was announced. They described the moment as overwhelming, with too much information delivered too quickly, leaving them feeling disoriented and unable to absorb key elements related to their treatment and prognosis.

“*I had a head-on shock because there was an avalanche of numbers. Surgeons, like oncologists, are immersed in their work*.” (Patient, FG5).

In contrast, those who received follow-up support from a nurse specialist immediately after the announcement found it highly valuable. This additional time allowed them to better process the information and ask questions at their own pace.

Family caregivers (FCs) often reported feeling excluded from the announcement process, despite being central to the support system. In some cases, the diagnosis was communicated abruptly and without sufficient emotional preparation, creating distress for both the person diagnosed and their close relatives.

### Stage 2: the care and treatment phase

#### Providing easy access to aervices

Most of the participants expressed concern about the amount of time they spent managing their medical appointments, including consultations and imaging. They indicated that administrative tasks took up a disproportionate amount of their time, to the detriment of their recovery. These administrative difficulties led to a feeling of abandonment by the healthcare system, a sense of having to fight their illness alone. Being connected to people who work in the health sector seems to make it easier to get medical or imaging appointments quickly..

“*My radiotherapy was 45 min away and no one helped me figure out how to get there. It was like: here’s your schedule, good luck*.” (Patient, FG2).

Some PCs and FCs highlighted a lack of information about available services, particularly supportive resources such as psychological support, social workers, or assistance for transport and housing.

#### Coordination of care

Participants highlighted a lack of coordination in most of cases, particularly between the various care establishments involved and with the general practitioner (GP). When this coordination is absent or inconsistent, the responsibility often falls on the patient, creating an additional burden.

In contrast, participants reported highly positive experiences when their healthcare establishment ensured effective coordination of their care, alleviating this responsibility and enhancing their overall experience.

“*I manage the various appointments with different specialists, and there is very little coordination between the services I need. For example, I have appointments with a surgeon specializing in bone tumors and with my oncologist, but they are in different hospitals. There’s no coordination between them; I’m the one who has to contact each of them one by one. When things don’t move quickly enough, I have to ask one to call the other, and so on, but it’s not done automatically*.” (Patient, FG2).

“*One doctor told me to stop the treatment, another said to continue. I didn’t know who to believe*.” (Patient, FG3).

#### Waiting for complementary tests

A source of major stress during this phase was the waiting period between initial exams and the start of treatment. Both PCs and FCs described long delays in accessing complementary or confirmatory tests, contributing to a heightened sense of vulnerability and fear.

“*The worst part wasn’t the diagnosis, it was waiting. Waiting to know. Waiting to start. Those weeks were unbearable*.” (Patient, FG4).

“*I can tell you they are extremely overloaded, and I’ve been waiting for two months to get this MRI done. My hematologist, who is actually the head hematologist at XXX, told me it’s best if I go somewhere else to get the MRI. So, they’re completely overwhelmed. It’s the same for PET scans, so I strongly advise you to look into another clinic*.” (Patient, FG1).

#### Supportive oncology care

Although supportive care is an integral component of the oncology care pathway [12], relatively few PCs reported having access to it. Many explained that they were not informed about available supportive services and often discovered them by chance or through informal channels, such as other PCs or FCs.

In most cases, supportive care was briefly mentioned during the diagnosis announcement consultation—a moment described as too emotionally overwhelming to absorb or process additional information.

Those who did access supportive care, however, described it as a deeply valuable part of their experience, providing both emotional support and practical tools to help navigate the treatment journey.

*I am here with my daughter, the psychologist comes, and the socio-esthetician comes, there are the nurses, so all of this is good because yes, we don't feel like we're in a hospital, getting treated, it's very family-like, we talk, there are massages. Well, there you go, it's said, it's great for that, it really helps me a lot too." (Patient, focus group 6)*.

#### Lack of humanity care and lack of availability of health professionals

Most participants highlighted the limited availability of healthcare professionals, particularly oncologists, noting that consultations were often too brief to address all their questions or provide comprehensive information about their treatment. Many felt they had to actively seek out information themselves.

Additionally, a significant number of participants reported a perceived lack of empathy from healthcare professionals, especially doctors. While they acknowledged and understood the heavy workloads faced by medical teams, they expressed regret over what they described as a dehumanization of care. Most of the PCs reported feeling alone and isolated during their treatment journey.

“*That feeling of humanity, which sometimes you feel is no longer there. You enter a professional medical protocol that sometimes leaves you a bit perplexed, and you think, 'Am I just a file? Where is the human aspect in all of this?*'” (Caregiver, focus group 3).

“*There are a lot of people, it's like a conveyor belt, but depending on how we are taken care of, sometimes we have the needle in the port-a-cath and our eyes don't even meet. […] Just a hello, how are you? Maybe we're recognized from one time to the next, or a kind word, it helps*.” (Patient, Focus-group 1).

### Stage 3: remission and recovery from cancer

The post-cancer period was often characterized by the participants as a profound sense of disappointment. While most participants expressed relief at having overcome the disease and avoided death, they faced an unexpected new challenge: coping with the aftermath of cancer. Many reported enduring physical after-effects such as persistent fatigue and residual pain. In addition to these physical challenges, this period required them to reassess their life goals and career plans, adding a layer of emotional and practical complexity to their recovery journey.

#### After-effects of cancer treatment

Many participants had to undergo long-term treatment during remission, or even for the rest of their lives. Managing the side effects of these treatments was an important aspect of their experience. While strong curative treatments and their side effects were often well accepted, those associated with treatments in remission were much less so. The participants indicated that the side effects had not been sufficiently explained or considered, even though they were likely to lead to significant impairments or even prevent people from returning to work or leading a ‘normal’ life.

*People say, 'Well, once you get through the chemo, everything will be fine. That's the hardest part,' and actually, that's not true at all. It's more afterward that's the problem. […] There's the hormone therapy, so you think it's over, but not at all, because it comes with a lot of side effects. There are absolutely monstrous musculoskeletal pains*.

#### Self-image and experience of cancer

Participants talked about the difficulty of accepting the distressing changes brought on by the disease. The lifestyle transition that they and their families had to go through was complex, as they needed to reappropriate their bodies after the various treatments. Several participants reported that the disease had affected their family and social relationships, forcing them to reorganise their personal and professional lives. In addition, the disease had sometimes led to financial difficulties, raising concerns about their current and future life plans.

“*I had this feeling of depersonalization, like they were taking a bit more of me. […] But if only we could avoid being reduced to just being a patient or feeling stripped of our charm and strengths*.” (Patient, FG4).

Many of the participants said that they had succeeded in adopting a philosophy of life that included integrative medicine, a holistic vision, physical activity or sport, and community involvement.

#### Returning to work

Returning to work was often seen as a sign of success in the face of illness by the participants. However, many participants had to adapt or even change their responsibilities due to the ongoing effects of their disease and treatment. Most of them expressed a desire for more support from their employers during this transition. At the same time, some saw it as an opportunity for retraining or greater job satisfaction.

“*It's also about professional retraining, and it’s very hard to have to fight, to say no. Just because I have cancer doesn’t mean I’ve lost my brain cells—I’m still capable of doing other things. Let’s try to figure something out together*.” (Patient, FG5).

At the same time, some saw it as an opportunity for retraining or greater job satisfaction.

#### Stage 3 the transition from curative to palliative care

Participants reported this period as marked by uncertainty and could occur while the PC remained relatively autonomous. PCs typically returned home with their FCs and often did not know where to seek help. Both PCs and FCs described feeling abandoned. FCs frequently reported a deep sense of helplessness, particularly those who were still employed. They described being left alone to manage pain relief, daily care, and emotional support, sometimes in the absence of the general practitioner.

Notably, none of the FCs who shared their experiences during this phase reported receiving support, despite significant psychological distress, which affected most of them. Additionally, FCs stated that they were not adequately supported after the death of their loved one.

“*But here we are, I’m alone with him. There’s no mobile team at home. I’m the one giving him his medications. There are no nurse visits, there’s absolutely nothing” (Caregiver, focus group 3)*.

“*It's complicated both for us as caregivers and for the person suffering from the condition because they are in psychological distress. This psychological distress is hard to assess, especially when the condition severely debilitates the person to the point where they can no longer express themselves or do anything*.”." (Caregiver, focus group 3).

“*They are alone with that, with this fear of death, with this end. We are heading toward something inevitable, and we don’t always feel supported and understood”. (Caregiver, focus group 3)*.

*When my loved one passed away, I felt completely abandoned: there was no support for us, the family. We were left alone to cope with our grief, utterly helpless. The bereavement leave, far too short, didn’t give me enough time to recover (Caregiver, focus group 3)*.

#### The beneficial role of patient associations in cancer care

During the focus groups, most participants highlighted the beneficial role of associations that provide essential support to PCs by providing administrative information and supportive care. They also organize activities that allow PCs to remain active in a suitable environment. A significant number of PCs and FCs became involved in an association after their illness and found it helpful in their social reconstruction. Participants highlighted the importance of making these associations better known from the beginning of the care pathway. The most frequently cited was “La Ligue contre le cancer.”

“*They are super nice. Do you know how much you pay per year? €20.00, and it's tax-deductible. You get a free hairdresser, free psychologist, free beautician services, and you can cook with them. Moreover, it's in a cozy environment with one or two dogs. The hairdresser is super nice. So, for €20.00 a year, you get all of this*.” (Patient, FG5.

## Discussion

This study offers valuable insights into the lived experiences of individuals with cancer (PCs) and their family caregivers (FCs) throughout the oncology care pathway. By focusing on their testimonies, it captures the emotional, physical, and logistical challenges they face, shedding light on gaps in the healthcare system that often go unnoticed. These findings emphasize the importance of placing patient and caregiver experiences at the center of oncology care policies and practices.

According to the literature, the diagnostic phase is one of the most emotionally challenging stages of the oncology care pathway, a finding echoed by both persons with cancer (PCs) and family caregivers (FCs) in our study. Many patients reported discovering signs of their illness during imaging or other complementary tests, even before receiving a formal diagnosis from a physician. This premature awareness, in the absence of adequate communication or explanation, was a significant source of anxiety and confusion.

These observations align with previous studies highlighting the psychological distress experienced by patients when radiological findings suggest cancer but are not promptly or clearly communicated. Leclère et al. and Ollivier et al. emphasized that radiologists often serve as the first point of contact with alarming results, yet their communication training is frequently insufficient for such sensitive discussions [13, 14]. Gutzeit et al. further argue that radiologists should reconsider their role in direct patient communication, as current practices may leave patients feeling isolated or inadequately informed during this critical moment [15].

Additionally, Grilo et al. demonstrated that imaging procedures themselves—particularly in oncology contexts—are associated with high anxiety levels, which often peak during the waiting period between the exam and formal diagnosis disclosure [16]. These findings underscore the importance of empathetic, timely communication and structured support during the diagnostic workup to reduce psychological burden and improve patient understanding.

According to the literature, when the cancer diagnosis is formally announced, patients and caregivers often experience this consultation as overwhelming. The volume and complexity of medical information, combined with the emotional impact of the news, can significantly impair the ability of persons with cancer (PCs) to process and retain key details about their condition and treatment options [17–19]. Jorda et al. [17] and Reich et al. [19] emphasized that inadequate communication strategies during diagnostic disclosure can deepen emotional distress and hinder comprehension. Similarly, Guerdoux et al. [18] demonstrated that poorly managed “bad news consultations” are associated with increased stress and a lower quality of patient experience.

However, the literature also indicates that follow-up interactions with trained oncology nurses can mitigate these effects. Redaelli et al. [20] and Meyer [21] highlighted the crucial role of nurse specialists and care coordination nurses in helping patients revisit and absorb essential information post-diagnosis. Their presence fosters a more personalized, empathetic dialogue and supports informed decision-making. Our findings confirm this, as participants who benefited from such follow-up reported a significantly more positive experience during this difficult phase.

These insights reinforce the need to institutionalize structured support systems involving trained nurse coordinators throughout the diagnostic and early treatment phases.

Yet in France, a funded system accompanied by official recommendations has existed since 2016, but it appears to have fallen short of its intended effectiveness [6]

According to literature, FCs often felt excluded from these discussions, further complicating their ability to support the patient effectively. These results reflect the deep emotional impact of the diagnosis phase on PCs and FCs and underscore the need for more empathetic and supportive communication during this critical moment [22–27].

Supportive care, a vital component of the oncology care pathway, was often underutilized according to participants’ experiences. Both PCs and FCs reported a lack of clear and accessible information about these services, leaving many to discover them by chance or through informal networks. This gap in communication meant that supportive care was often overlooked, despite its potential to significantly enhance the patient journey.

The testimonies of PCs and FCs revealed a troubling sense of disconnection from healthcare professionals. Many participants felt that the relational aspect of care was often overshadowed by the clinical and procedural demands of oncology treatment. Short consultations and the limited availability of oncologists left patients feeling rushed and unable to ask all their questions or address their concerns. As a result, they frequently had to seek out information on their own, adding to their emotional burden.

The perceived lack of empathy from healthcare professionals further deepened these feelings of isolation. While participants acknowledged the heavy workloads faced by medical teams, they expressed regret over what they described as a "dehumanization" of care. These experiences highlight the critical need for healthcare professionals to balance clinical efficiency with emotional support to ensure a more compassionate approach to patient care. However, numerous studies have shown that physicians' empathy, particularly as perceived by patients, is associated with better outcomes [27–32].

Effective care coordination was a major concern raised by participants, particularly during the transition to palliative care. Individuals with cancer (PCs) and their family caregivers (FCs) described difficulties navigating a complex system where the lack of coordination between hospital specialists and general practitioners (GPs) often resulted in a sense of abandonment. These findings are consistent with Zøylner et al. [22], who highlighted the unmet needs of surgical breast cancer patients and their relatives due to poor coordination and lack of clarity in the care pathway. Similarly, Baudry et al. [23] demonstrated that emotional distress and unmet informational needs among caregivers were strong predictors of reduced quality of life.

These organizational challenges were compounded by limited access to supportive care. Many participants reported only learning about available services by chance, echoing systematic reviews that underline persistent gaps in access to and communication about supportive care for patients with advanced cancer and their caregivers [25, 26]

Moreover, the emotional burden and lack of structured support for caregivers were prominent in our study. Oberoi et al. [24] found that unmet informational needs and negative care experiences among caregivers were associated with significantly higher levels of anxiety and depression.

The lack of time and attentiveness from healthcare professionals also contributed to a perceived “dehumanization” of care. This concern is well documented in the literature. Numerous studies have demonstrated that physician empathy, particularly as perceived by patients, is associated with better outcomes in oncology care [27]. Additionally, recent research shows that the way “bad news” is delivered significantly influences patients’ perception of empathy during consultations [28], and that teaching relational “power skills” can improve physician self-efficacy, reduce burnout, and enhance patient outcomes [29].

Finally, systemic constraints—such as the short consultation times in French general practice, as highlighted in international comparisons [30] and national surveys [31]—hinder the ability to provide comprehensive, individualized care. Yet cancer patients’ preferences for receiving bad news are clear: they expect time, attentiveness, and a personalized communication style [32].

Participants also noted that supportive care was typically introduced during the announcement consultation, a moment already saturated with information and emotions. This timing limited patients’ ability to fully understand its importance or access its benefits. However, those who did engage with supportive care described it as a transformative and invaluable resource, illustrating it’s potential when effectively integrated into the care pathway.

The transition to palliative care was especially challenging for family caregivers (FCs), who often found themselves shouldering significant caregiving responsibilities with minimal guidance or institutional support. Participants described intense feelings of helplessness and frustration, particularly when pain management for the person with cancer (PC) was inadequate or poorly coordinated. This experience mirrors findings from Zøylner et al. [22], who documented similar unmet needs among caregivers during critical phases of cancer treatment.

Additionally, several studies have emphasized that the lack of systematic support structures leaves caregivers emotionally and practically overwhelmed [33]. Zimmermann et al. [34] found that both patients and caregivers frequently misunderstood the purpose and timing of palliative care, contributing to delays in accessing necessary services. Reblin et al. [35] and Sklenarova et al. [36] reported that caregivers face high levels of anxiety and depression, especially when their role is not recognized as part of the healthcare process. Teixeira et al. [37] further demonstrated the psychophysiological toll of prolonged caregiving without adequate institutional support.

Our study supports these findings and highlights an urgent need for the better integration of palliative care services and targeted caregiver support, especially during the transition phase. Without such measures, FCs are left to navigate emotionally complex and medically demanding situations on their own, often at great personal cost.

The post-cancer period, while a time of relief for many, introduced new challenges that were often overlooked by the healthcare system. PCs frequently reported persistent physical side effects, such as chronic fatigue and pain, which significantly impacted their quality of life and ability to return to normalcy. This phase also required patients to reevaluate their life and career plans, a process that many found overwhelming without adequate support.

For FCs, the post-cancer period brought additional emotional and psychological burdens, particularly after the loss of their loved ones. Many caregivers described feeling abandoned and unsupported during their bereavement, these findings echo those of previous studies, which have also underlined the lack of structured support for caregivers during the grieving process and the need for targeted resources to address their specific challenges [38].

The experiences of PCs and FCs emphasized the critical role of supportive services, such as those provided by patient associations like "La Ligue contre le cancer" [39]. These organizations were praised for offering administrative, emotional, and practical support. However, participants noted that these services were not well-integrated into the care pathway and were often underutilized due to a lack of awareness.

The testimonies suggest that systematically incorporating supportive services from the moment of diagnosis could significantly improve the overall care experience. By addressing both the practical and emotional needs of patients and their caregivers, these organizations can play a vital role in enhancing oncology care.

Over successive national cancer plans, the integration of patient experience has gained growing recognition. Several initiatives have supported the emergence of "patient resource" roles to assist individuals affected by cancer. These developments are in line with other studies that have highlighted the increasing institutional attention to patient involvement. However, in the "Ten-Year Strategy for Fighting Cancer 2021–2030," [8] the terms "patient experience" and "patient partner" are not explicitly mentioned. Nonetheless, the strategy adopts a patient-centered approach, particularly through Axis 2, which prioritizes reducing treatment-related sequelae and improving quality of life. As other authors have argued, a more explicit formalization of patient roles could further promote direct involvement of patients and caregivers in the co-construction of care pathways [8].

*Limitations to research* some limitations should be considered when interpreting the results. It is possible that a self-selection bias influenced the sample, particularly if individuals who experienced more difficult or impactful situations were more likely to participate. However, the diversity of testimonies collected—including a wide range of experiences—suggests that the data remain representative of varied situations. Additionally, recall bias may have affected some accounts. Nevertheless, the participants’ narratives, often expressed with clarity and emotional depth, indicate that the recollections retain strong qualitative value. Finally, other common limitations of qualitative research apply, including the transferability of findings to other contexts, although the triangulation of perspectives between patients and family caregivers helps to enrich the overall understanding of the post-cancer journey.

### Implications for practice

For Corse and PACA region, this study has led to the implementation of an audit, currently underway, conducted by the PACA CORSE regional oncology network. The audit focuses on whether the announcement consultation is carried out in accordance with guidelines from the perspective of healthcare professionals, as well as on the patient experience.

In addition, as part of a broader initiative to recognize the role of patients and users, the Regional Health Agency (ARS) of PACA has established a regional “Patient Experience” group. This group brings together healthcare professionals, patient associations, and patients themselves, with the goal of more effectively integrating the voice of patients into the regional organization of care. It provides a space for dialogue and co-construction, helping to position patient experience as a lever for transforming the healthcare system.

### Recommendations to improve the oncology pathway

The findings of this study, rooted in the lived experiences of PCs and FCs, highlight several areas for improvement:

- Rethink the diagnosis process: Ensure that both PCs and FCs are actively involved in announcement consultations and provide follow-up discussions with specialist nurses to support understanding and decision-making.
- Enhance access to supportive care: Provide clear and timely information about supportive services at appropriate stages of the care pathway to maximize their impact.
- Prioritize human connection: Train healthcare professionals in empathetic communication and allocate sufficient time for consultations to address patients’ concerns comprehensively.
- Strengthen care coordination: Foster collaboration between GPs and specialized teams to ensure seamless transitions and reduce the burden on patients and caregivers.
- Support post-cancer needs: Develop dedicated services to manage long-term side effects, facilitate professional reintegration, and provide bereavement counselling for FCs.
- Integrate patient associations: Systematically incorporate these organizations into the care pathway to provide continuous and accessible support.

## Conclusion

This study, grounded in the lived experiences of PCs and FCs, underscores the profound impact of cancer on every aspect of life. From diagnosis to post-cancer recovery, the findings reveal significant gaps in care delivery, coordination, and emotional support. Addressing these issues requires a systemic shift toward patient-centered care that prioritizes the voices and experiences of patients and their caregivers.

By placing the lived experiences of PCs and FCs at the heart of oncology care policies and practices, the healthcare system can build a more compassionate, effective, and supportive framework for those navigating the complexities of cancer.

## Data Availability

No datasets were generated or analysed during the current study.
